# Fast 3D shape screening of large chemical databases through alignment-recycling

**DOI:** 10.1186/1752-153X-1-12

**Published:** 2007-06-06

**Authors:** Fabien Fontaine, Evan Bolton, Yulia Borodina, Stephen H Bryant

**Affiliations:** 1 National Center for Biotechnology Information, National Library of Medicine, National Institutes of Health, Department of Health and Human Services, 8600 Rockville Pike, Bethesda, MD 20894, USA

## Abstract

**Background:**

Large chemical databases require fast, efficient, and simple ways of looking for similar structures. Although such tasks are now fairly well resolved for graph-based similarity queries, they remain an issue for 3D approaches, particularly for those based on 3D shape overlays. Inspired by a recent technique developed to compare molecular shapes, we designed a hybrid methodology, alignment-recycling, that enables efficient retrieval and alignment of structures with similar 3D shapes.

**Results:**

Using a dataset of more than one million PubChem compounds of limited size (< 28 heavy atoms) and flexibility (< 6 rotatable bonds), we obtained a set of a few thousand diverse structures covering entirely the 3D shape space of the conformers of the dataset. Transformation matrices gathered from the overlays between these diverse structures and the 3D conformer dataset allowed us to drastically (100-fold) reduce the CPU time required for shape overlay. The alignment-recycling heuristic produces results consistent with *de novo *alignment calculation, with better than 80% hit list overlap on average.

**Conclusion:**

Overlay-based 3D methods are computationally demanding when searching large databases. Alignment-recycling reduces the CPU time to perform shape similarity searches by breaking the alignment problem into three steps: selection of diverse shapes to describe the database shape-space; overlay of the database conformers to the diverse shapes; and non-optimized overlay of query and database conformers using common reference shapes. The precomputation, required by the first two steps, is a significant cost of the method; however, once performed, querying is two orders of magnitude faster. Extensions and variations of this methodology, for example, to handle more flexible and larger small-molecules are discussed.

## Background

Databases of chemical structures are a key component of chemical information infrastructures. Searching these databases requires specialized methods, for example, to find similar chemical structures.

There are many ways [1-4] to define "similarity" between chemical structures. Generally, chemical similarity is determined by comparison of "fingerprints" using the Tanimoto equation (**Eq. 1**). The fingerprints are often binary bit strings with each set bit, or pattern of set bits, representing the presence of a particular topological fragment in a molecule.

**Figure 1:**
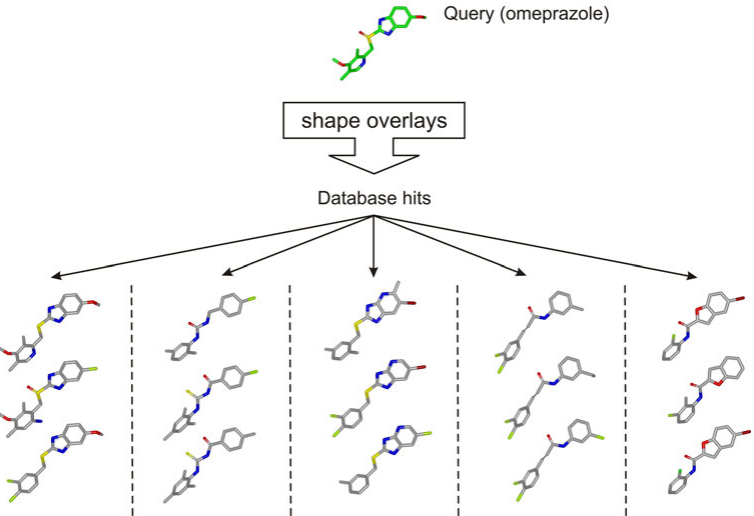
Examples of output from a shape search using the proton pump inhibitor omeprazole on a subset of PubChem organized by structural class.

Tanimoto=ABA+B−AB
 MathType@MTEF@5@5@+=feaafiart1ev1aaatCvAUfKttLearuWrP9MDH5MBPbIqV92AaeXatLxBI9gBaebbnrfifHhDYfgasaacH8akY=wiFfYdH8Gipec8Eeeu0xXdbba9frFj0=OqFfea0dXdd9vqai=hGuQ8kuc9pgc9s8qqaq=dirpe0xb9q8qiLsFr0=vr0=vr0dc8meaabaqaciaacaGaaeqabaqabeGadaaakeaacqWGubavcqWGHbqycqWGUbGBcqWGPbqAcqWGTbqBcqWGVbWBcqWG0baDcqWGVbWBcqGH9aqpdaWcaaqaaiabdgeabjabdkeacbqaaiabdgeabjabgUcaRiabdkeacjabgkHiTiabdgeabjabdkeacbaaaaa@40B7@

where *AB *is the count of common set bits and *A *and *B *are the count of set bits Similarity measures of this type make it possible to perform searches of chemical databases, containing millions of compounds, in a matter of seconds. While fast, these "2D similarity" methods tend to prefer compounds of similar structural class or topology as the query; however, "3D similarity" methods use geometric constraints and are valued for their ability to find compounds belonging to diverse chemical families [5] (Figure 1).

The computational cost of 3D methods, however, is dramatically greater than 2D methods, due to the relative complexity of generating, selecting, and comparing various 3D representations of chemical structures. The cost is particularly severe when the comparisons are done by structural overlay, when considering the additional step of determining an optimal 3D overlay. As such, some groups have focused, for example, on extending 2D methods for the discovery of topologically non-obvious similar compounds using reduced-graph approaches [6-8]. With the increase of available computer power, fast 3D structural overlay software, such as ROCS [9], has become attractive for large database screening.

ROCS performs rapid overlays of 3D chemical structures using atom-centered Gaussians to compute geometric overlap [10]. Similarity is measured with the shape Tanimoto equation (**Eq. 2**); unlike 2D, an estimate of molecular volume overlap is used, instead of bit counts.

shapeTanimoto=OABOA+OB−OAB
 MathType@MTEF@5@5@+=feaafiart1ev1aaatCvAUfKttLearuWrP9MDH5MBPbIqV92AaeXatLxBI9gBaebbnrfifHhDYfgasaacH8akY=wiFfYdH8Gipec8Eeeu0xXdbba9frFj0=OqFfea0dXdd9vqai=hGuQ8kuc9pgc9s8qqaq=dirpe0xb9q8qiLsFr0=vr0=vr0dc8meaabaqaciaacaGaaeqabaqabeGadaaakeaacqWGZbWCcqWGObaAcqWGHbqycqWGWbaCcqWGLbqzcqWGubavcqWGHbqycqWGUbGBcqWGPbqAcqWGTbqBcqWGVbWBcqWG0baDcqWGVbWBcqGH9aqpdaWcaaqaaiabd+eapnaaBaaaleaacqWGbbqqcqWGcbGqaeqaaaGcbaGaem4ta80aaSbaaSqaaiabdgeabbqabaGccqGHRaWkcqWGpbWtdaWgaaWcbaGaemOqaieabeaakiabgkHiTiabd+eapnaaBaaaleaacqWGbbqqcqWGcbGqaeqaaaaaaaa@4CF0@

where *O*_*AB *_is the volume overlap between conformer *A *and conformer *B*, *O*_*A *_is conformer *A *volume, and *O*_*B *_is conformer *B *volume

Several published applications of ROCS demonstrate its usefulness in practical medicinal chemistry projects [11-13]. ROCS can screen the dataset used in this work at the rate of ~1 800 conformers per second per (64-bit 3-GHz Intel dual core Xeon) processor. Although this is a remarkable speed for this kind of software, it can still take hours to perform a single search of a moderately sized 3D database containing millions of conformers.

Innovative overlay-based approaches [14,15] have been created to avoid brute-force comparison between a query conformer and each and every conformer in a 3D database. One approach [15] involves finding a small "dictionary" of 3D structures that represent the overall diversity of possible 3D shapes. These diverse shapes are then used to create a binary "3D fingerprint" for each conformer in a database, with each set bit corresponding to a computed similarity above a predefined threshold between the diverse shape and the database conformer. This technique shifts the substantial 3D computational overhead into the initial selection of diverse shapes and the generation of the 3D fingerprint for all conformers in the database. For each 3D similarity query, the workflow now becomes identical to that of 2D binary fingerprint methods: compute the fingerprint for the query; loop over the database contents; and determine the bits in common for computation of **Eq. 1**. After a 3D fingerprint is designed and created, such an approach can significantly reduce the time to search moderately sized 3D databases, *e.g*., by shape similarity, from hours to minutes.

There are two major differences between the results from brute-force ROCS shape overlay comparison and the 3D shape fingerprint [15] similarity method. Firstly, the two methods use very different measures for the Tanimoto values and are not guaranteed to give similar results. Secondly, the 3D shape fingerprint similarity approach does not provide a 3D alignment with the query, thus making the results difficult to analyze or visualize. In this study, we attempt to modify an earlier 3D shape similarity approach [15] to mimic results provided by brute-force ROCS similarity searching, but at a fraction of the computational expense. A novel aspect of our method, which we call "alignment-recycling", comes from recycling the translational and rotational matrices resulting from the shape overlay during the initial selection of diverse shapes.

## Results

### Subset extraction

At the time of project initiation, the PubChem Compound [16] database contained approximately 5.3 million unique chemicals and mixtures. We focused our attention on a subset of PubChem by targeting only single-component molecules with size and flexibility below lead-like [17] or drug-like [18] filtering cut-offs. Our strategy was to work with a simple but relevant subset that could be incrementally updated with more challenging compounds in future studies.

**Figure 2:**
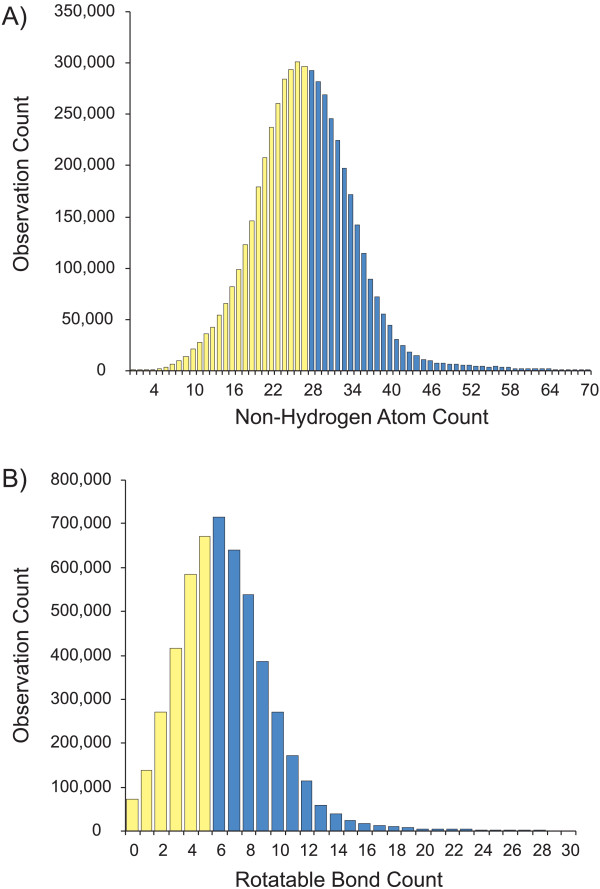
**Distribution of PubChem single component compounds**. **A) **According to number of heavy atoms. **B) **According to number of rotatable bonds. Yellow bars represent the range of compounds included in the dataset. For a better scaling of the histograms, covalent units above 70 heavy atoms and above 30 rotatable bounds were excluded from the plots.

**Figure 3:**
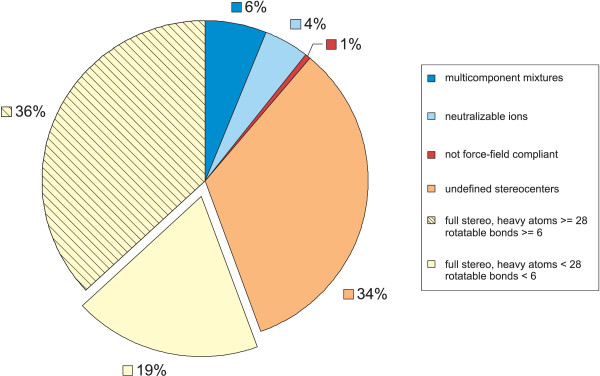
Selection of study compound subset from the entire PubChem Compound database.

The distributions of non-hydrogen (heavy) atoms and rotatable bonds for PubChem single component structures are presented in Figure 2. For this study, we limited our work to the first half of each distribution, *i.e*., just those small molecules (less than twenty-eight non-hydrogen atoms) with low flexibility (less than six rotatable bonds). Furthermore, we removed all compounds with incomplete stereochemistry (stereo atoms or bonds), to avoid enumerating multiple stereo-configurations. We also removed ionic forms of structures, since their neutralized forms will be contained in the PubChem compound dataset. The PubChem compound subset selected is summarized in Figure 3. Despite restrictions, the final dataset resulted in approximately one million (1 035 040) unique PubChem compounds representing about 19% of PubChem at the time of dataset extraction. Most of the structures are drug-like organic compounds and, therefore, are well suited for the MMFF94s force field [19] implemented in the 3D conformer generator OMEGA 1.8.1 and 2.0 Beta [20] used for this study.

### Algorithms

The alignment-recycling (**AR**) methodology is intended to obviate performing the optimization required to maximize the volume overlap of the query conformer to each and every conformer in a 3D conformer dataset. This is achieved by selecting representative conformers to completely cover the "shape space" of the 3D conformer dataset. The granularity of coverage is defined by an empirical cutoff named "Design-Tanimoto" (see section **Reference shape selection**). Each conformer in the dataset is overlaid to each representative conformer and the overlay information is retained, if the similarity with a representative conformer is of sufficient magnitude.

The empirical criterion to decide if two overlaid conformers can be considered similar is named "Transform-Tanimoto" (see section **Alignment recycling**). Its value greatly influences the number of reference shapes associated with each conformer. By means of analogy to a binary fingerprint, the Transform-Tanimoto threshold defines when a bit is set.

To search the dataset by shape similarity, the query fingerprint, to extend the analogy, is compared to the dataset fingerprints to find common reference shapes. The Tanimoto value computed between query and database fingerprints with **AR **is not that from **Eq. 1**, as is typical with 2D fingerprint methods and used by the 3D fingerprint method of Haigh *et al*. [15]. Instead, finding a common reference shape triggers computing, via **Eq. 2**, the shape Tanimoto between the query conformer and database conformer, as may be performed by a typical brute-force ROCS approach. In our method, the 3D conformer overlay used in computing the shape Tanimoto is generated by simply reusing the transformation, *i.e*., the rotation matrix and translation vector, from the overlay to the common reference shape. This trivial transformation, while specific to alignment of a reference conformer, when applied, can yield a relatively accurate shape overlay between the query and database conformers without the need to perform the conformer overlay alignment optimization. Usually, when the query and a database conformer are fairly similar, multiple reference shapes are found to be in common. In such cases, all reference shape alignments are reused to find a maximum shape Tanimoto between conformers.

### Reference shape selection

As described in the **Method **section, we implemented the clustering algorithm of Haigh *et al*. [15] to select a diverse set of reference shapes. For this study, we chose a Design-Tanimoto value of 0.75, which, according to their work, represented the best trade-off between sampling speed and granularity. This means, by definition, no pair of reference shapes has a similarity above 0.75, after diversity selection, and that every conformer in the entire dataset is associated with at least one reference shape with a shape Tanimoto similarity above 0.75.

Diverse reference shape selection for the one million compound dataset was performed in two stages. In the first stage, only a single conformer representative generated by OMEGA 1.8.1 [20] was used. The single conformer dataset was entirely covered after the inclusion of 2 458 reference shapes. In the second stage, we sampled the conformational space of each compound using OMEGA 2.0 Beta[20] at an RMSD of 1.0 Å. This generated approximately fifteen million (14 925 817) conformers. The distribution of conformers per compound is strongly skewed towards low values, with 50% of the compounds having six or fewer conformers and only 10% of the compounds accounting for 49% of the total conformer count. Interestingly, 99.8% of the fifteen million conformers in the second stage can be clustered at a Design-Tanimoto of 0.75 using one of the initial 2 458 reference shapes of the single conformer subset, revealing a large amount of shape redundancy in the multi-conformer models. However, the shape space of the remaining 0.2% conformers increases the number of diverse reference shapes from 2 458 to 5 534. This potentially surprising result may be a consequence of the sphere-exclusion algorithm variant used for the reference shape selection. In the attempt to cover the entire dataset shape space with a minimum number of reference shapes, the algorithm tends to leave 'holes' in the shape space, thus producing unequally sampled regions. Given the substantial redundancy of conformer shapes in the multi-conformer model dataset, it is very likely that a large fraction of the additional 3 076 reference shapes is necessary to fill these holes. There is no direct indication that the additional reference shapes resulted from anything more than sampling deficiency, *i.e*., were not directly attributable to the size or flexibility of molecules. Use of more efficient sampling algorithms designed to avoid empty spaces, *e.g*., DISE [21], may lead to more efficient shape space coverage than that used in this study.

Because we aim at selecting a diverse set of shapes, the reference conformers appear to represent particular structural features to a greater extent than are present in the entire dataset. For example, only 20% of the PubChem dataset contain chiral centers; however, 33% of the reference shapes contain a chiral center. Similarly, a (non-exhaustive) trend is found between the dataset and reference conformers for triple bonds (8% versus 37%), lack of aromatic atoms (6% versus 21%), and presence of a ring system with more than six atoms (3% versus 28%). As a consequence, reference shapes generated from structures with less common features tend to cluster fewer database conformers than those coming from compounds with more common features.

### Alignment-recycling (AR)

**Figure 4:**
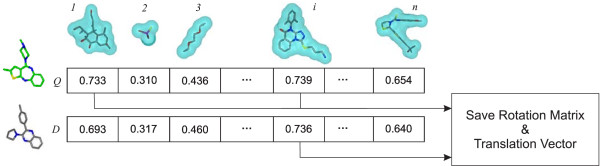
**Conformer alignment to reference shapes**. *Q *is the query conformer. *D *is the dataset conformer. Reference shapes are numbered from *1 *to *n*. Any alignment with a shape Tanimoto above the Transform-Tanimoto value of, in this case, 0.73 is stored for reuse during database screening.

**AR **takes advantage of information created upon comparison of the reference shapes to a conformer during shape fingerprint generation. When a conformer is overlaid on a reference shape, and the computed shape Tanimoto is above the Transform-Tanimoto, the data required to reproduce that alignment are saved (Figure 4). Such information has the form of a three-by-three rotation matrix and a translation vector. In contrast to ROCS, **AR **can only occur when the query conformer structure is found to have a reference shape in common with a database conformer. The alignment between the query conformer and database conformer is determined using the retained rotational matrices and translational vectors relative to that reference shape.

**Figure 5:**
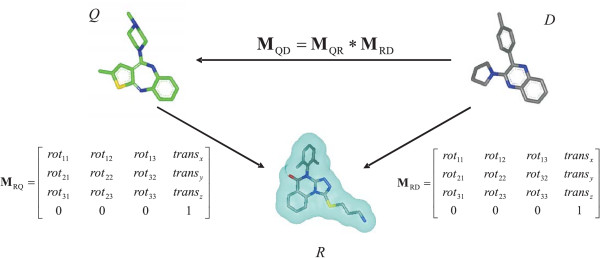
**Alignment recycling**. *Q *is the query conformer. *D *is the dataset conformer. *R *is the reference shape. **M**_QD _is the 4 × 4 matrix used to align *D *onto *Q*. **M**_QD _is calculated on the fly through the product of the pre-computed query/reference (**M**_QR_) and reference/dataset (**M**_RD_) alignment matrices.

The procedure to align the query conformer, *Q*, and database conformer, *D*, is the following (as depicted in Figure 5). The three-by-three rotation matrix and the translation vector to overlay the database conformer *D *on the reference shape *R *are merged into a single four-by-four affine transformation matrix (**M**_**RD**_). Similarly, one can construct the four-by-four affine transformation matrix **M**_**QR **_by using the transpose of the three-by-three rotational matrix and the minus of the translational vector from the overlay of the query conformer *Q *on the reference shape *R*. The matrix **M**_**QD **_is produced by the matrix multiply of **M**_**QR **_with **M**_**RD**_. Conformer *D *is aligned on conformer *Q *by multiplying the coordinate vector of each atom of *D *with **M**_**QD**_. In some aspects, the method is conceptually similar to structural alignments performed in 3D-QSAR methodologies for which all the conformers of the dataset are aligned on the same reference template. In our case, the reference template is a reference shape pre-selected during the initial diverse selection.

Each time an alignment is attempted after transformation matrices combination, the quality of the alignment is evaluated by a single point shape Tanimoto estimation via a Gaussian Grid approximation similar to ROCS, as detailed in the **Methods **section. The final number of matrix multiplications and alignments depends on the Transform-Tanimoto value as well as the number of reference shapes in the vicinity of the query conformer.

In practice, a single combination of transformation matrices cannot guarantee a result close to an optimal structural alignment. Some conformers may have different optimal alignments with a reference shape due to structural symmetry; however, the presence of multiple reference shapes greatly increases the chance of finding an alignment very close to the analytical maximum overlap solution. A convenient property of the method is that similar structures tend to have more reference shapes in common than dissimilar ones, thus far more CPU time is dedicated to the alignment of similar structures than for dissimilar structures.

### Finding the right Transform-Tanimoto

Similarity searches often require a threshold as a simple criterion to prune the hit list. The threshold value is somewhat subjective although a reasonable range of useful values can be deduced from the literature involving ROCS. Rush *et al*. [11] mention a general rule-of-thumb that a shape Tanimoto value greater than 0.75 provides visual shape similarity, although they used a 0.85 threshold to select their ZipA-FtsZ protein-protein inhibitors. According to a regression plot from Bostrom *et al*. [22] and our own in-house experience, a RMSD cut-off of 1.0 Å used during conformational sampling with OMEGA 2.0 Beta roughly corresponds to a shape Tanimoto between 0.75 and 0.85. In their virtual screening study, Muchmore *et al*. [13] found a melanin-concentrating hormone receptor 1 antagonist with nanomolar IC50 at a shape Tanimoto above 0.80. Taking these studies into account, our range of interest in finding similar shapes is limited to ROCS shape Tanimoto between 0.75 and 1.0, alignments with lower similarity values were not considered for this work.

The suitable Transform-Tanimoto value, which determines if two structures have a reference shape in common and enables alignment via matrix multiplication, was determined empirically. For that, we performed a set of random overlays using both ROCS and alignment-recycling. Our objective was to keep the Transform-Tanimoto value as high as possible to limit the possible number of matrix combinations and, in doing so, save substantially on CPU time. We started by setting the Transform-Tanimoto value to the Design-Tanimoto value, *i.e*., 0.75. When applying the **AR **technique, alignment cases where two conformers do not share a common reference shape are assigned a shape Tanimoto value of zero. Because the initial Transform-Tanimoto threshold was not providing the quantity of hits to be consistent with the brute-force approach, primarily due to not finding appropriate reference shapes in common, we progressively decreased the Transform-Tanimoto value by 0.01.

**Figure 6:**
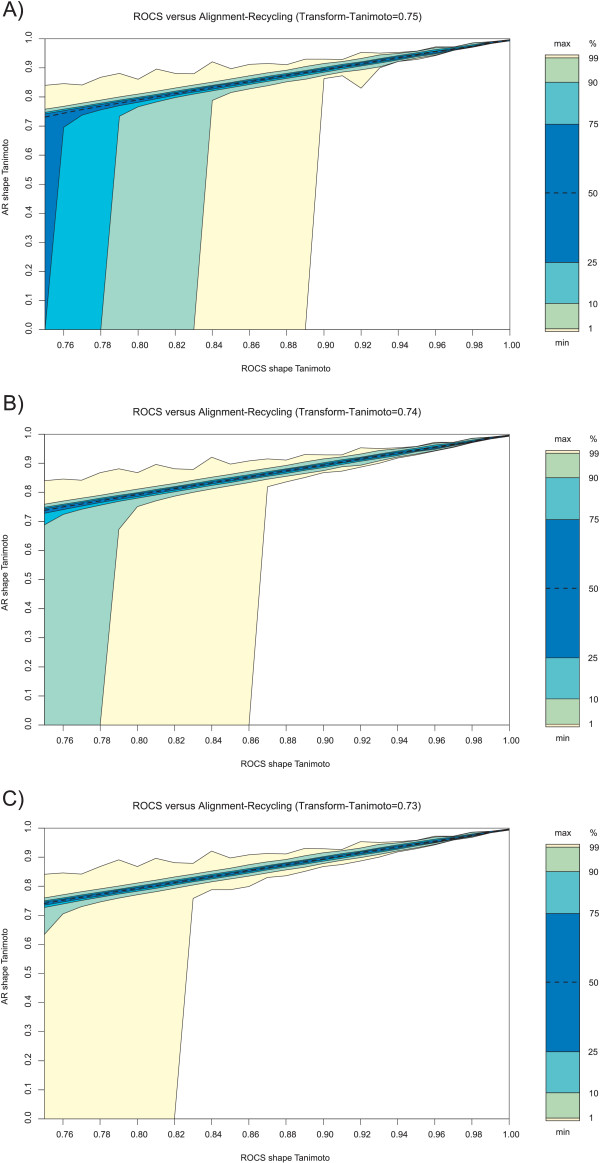
**ROCS versus alignment-recycling (AR) shape Tanimoto**. **A) **Transform-Tanimoto equal to 0.75. **B) **Transform-Tanimoto equal to 0.74. **C) **Transform-Tanimoto equal to 0.73. The quality of the correlation improves as the Transform-Tanimoto threshold is decreased. Isocontours represent the distribution of **AR **alignments for each 0.01 ROCS shape Tanimoto interval. Distribution is successively partitioned at first percentile, first decile, first quartile, median, last quartile, last decile and last percentile. The scale on the side of each plot is proportional to the number of alignments in each partition.

The relation between ROCS and alignment-recycling at several Transform-Tanimoto values is plotted on Figure 6. The plots are based on ~1.3 million (1 283 211) alignments with a ROCS shape Tanimoto in the range 0.75–1.0. This subset is part of a training set of thirty million random shape-overlays, generated by comparing 2 000 random conformers against 15 000 random conformers from the fifteen million PubChem conformer dataset. The particular nature of the distribution is unveiled by binning the data every 0.01 shape Tanimoto and plotting the isocontour lines at commonly used thresholds for proportion estimation. The scale on the side of each plot gives an indication of the proportion of the data points between each isocontour. All the data points are contained between the minimum and the maximum of each bin, the other isocontour lines (i.e. first and last percentile, decile, and quartile) highlight the intrinsic distribution of the data among each bin. The plots indicate that there is no hard shape Tanimoto limit between finding and not finding a common reference shape between ROCS and **AR**, but rather some probabilistic distribution. For example, at 0.75 Transform-Tanimoto, 25% of the alignments with a 0.75 ROCS shape Tanimoto do not share an associated **AR **reference shape. This proportion decreases to less than 10% at a Transform-Tanimoto of 0.74, and less than 1% at 0.73. By means of comparison, an **AR **reference shape is always found in common for the Transform-Tanimoto values 0.75, 0.74, and 0.73 at ROCS shape Tanimoto values of 0.89, 0.86, and 0.82, respectively. Although we could have further decreased the Transform-Tanimoto to even lower values, in order to further decrease or eliminate the chance of not finding an **AR **reference shape in common, we felt that a Transform-Tanimoto equal to 0.73 produced satisfying results.

**Figure 7:**
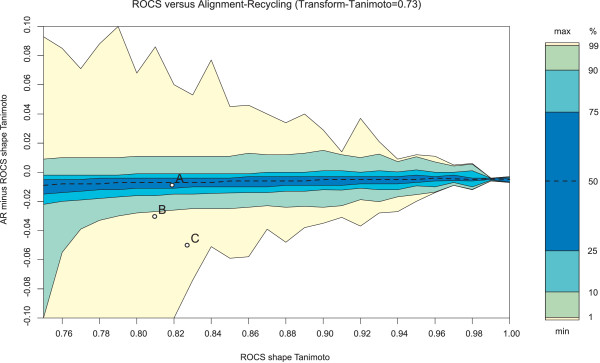
**Alignment-recycling (AR-0.73) minus ROCS shape Tanimoto**. Isocontours represent the distribution of shape Tanimoto differences between **AR-0.73 **and ROCS alignments for each 0.01 ROCS shape Tanimoto interval. **AR-0.73 **performs better than ROCS when the difference is above 0, and vice-versa. Distribution is successively partitioned at first percentile, first decile, first quartile, median, last quartile, last decile and last percentile. The scale on the side of each plot is proportional to the number of alignments in each partition. Points A, B and C correspond to the examples shown in **Figure 8**.

**Figure 8:**
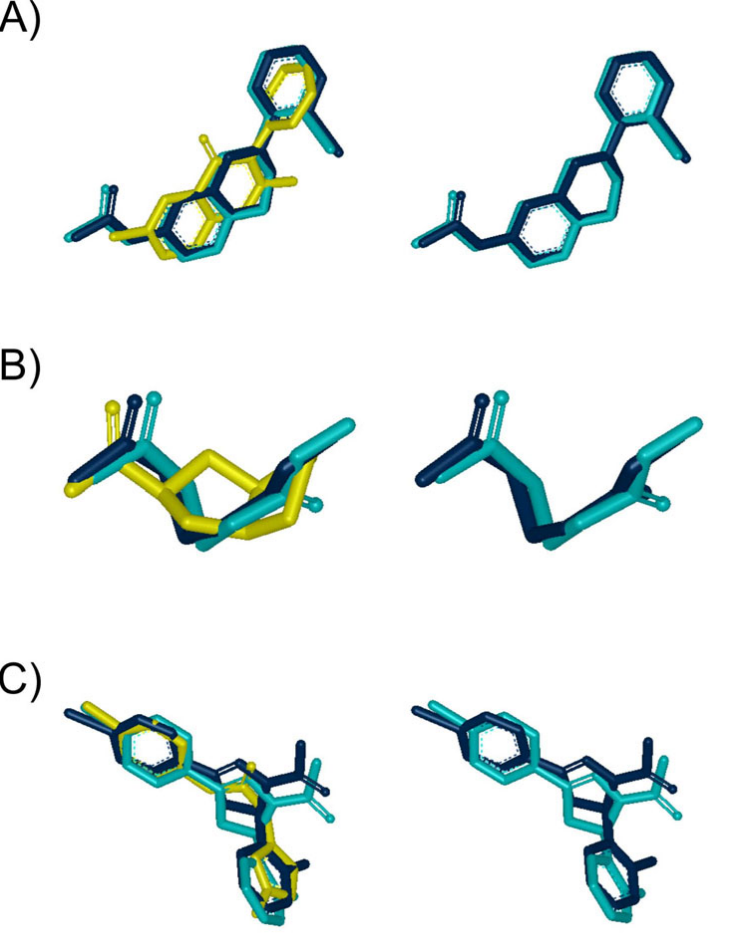
**Impact of lower alignment quality**. Examples of ROCS versus **AR-0.73 **alignments from **Figure 7**. Left side: Query (yellow), ROCS alignment (dark blue) and **AR **alignment (cyan). Right side: Same as left side but with query removed.**A) AR **shape Tanimoto 0.01 worse than ROCS. **B) AR **shape Tanimoto 0.03 worse than ROCS. **C) AR **shape Tanimoto 0.05 worse than ROCS.

A more detailed comparison on the relative performance of **AR **at 0.73 Transform-Tanimoto is plotted on Figure 7. A negative difference shows that the ROCS overlay is better than the recycled overlay. In contrast to ROCS, **AR **does not aim at finding the exact global alignment solution. Instead, **AR **attempts to provide an alignment that is very close with little difference in terms of the shape Tanimoto and graphical display. Consequently, **AR **overlays are 0.01 shape Tanimoto less than the ROCS overlays 25% of the time (Figure 7: point A); however, this difference is visually minor as shown in Figure 8a. A decrease of 0.03 shape Tanimoto (Figure 7: point B), as shown in Figure 8b, brings some visual separation to the **AR **and ROCS alignments. At a difference of 0.05 shape Tanimoto (Figure 7: point C), there is a clear visual difference between the overlays, although they are still qualitatively the same (Figure 8c). The degree of alignment quality that may be required by a user depends strongly on the intended use of the alignment, and **AR **alignments could certainly be used as a very good starting point for subsequent shape overlap optimization, *e.g*., using ROCS.

The distribution in Figure 7 also indicates that about 1% of the time alignment-recycling performs relevantly better (shape Tanimoto difference > 0.01) than ROCS. One possible explanation for this observation is that ROCS gets locked into a local minimum during overlap optimization. A more likely explanation is differences in the numerical precision of the ROCS *Grid *method versus ours (see section **Gaussian shape overlay**). Overall, the chance of getting a poor **AR **alignment, as compared to one produced by ROCS, is relatively rare, when using a 0.73 Transform-Tanimoto value and considering the full 0.75–1.0 shape Tanimoto range.

### Comparing speed and hit lists

The test set used here for speed and hit list comparison contained 65 compounds extracted from a dataset of leads and drugs from Oprea *et al*. [17]. Each test set compound was represented by a single random low-energy conformer. Together, the 65 conformers span a diverse range of shapes derived from simple structures, *e.g*., salicylic acid, to fairly complex ones, *e.g*., morphine. The CPU time required to query the fifteen million conformer dataset using the various methods is shown in Table 1. To compute ROCS shape overlays for the entire conformer dataset takes, on average, 2.3 hours, while the time required to perform **AR **screening at 0.73 Transform-Tanimoto is about 1.3 minutes. This represents more than a 100-fold speedup.

**Table 1:** CPU time to query the fifteen million conformer database with a single conformer

**Method**	**CPU Time (min.)**		
	
	**Average**	**Minimum**	**Maximum**
*ROCS*	136	113	216
*AR-0.73 (Total)*^a^	1.28	0.33	3.32
*AR-0.73 (Screening part)*	1.11	0.17	3.13
*AR-0.74 (Screening part)*	0.65	0.15	1.85
*AR-0.75 (Screening part)*	0.38	0.11	1.15
*Query vs. 5 534 AR Reference Shapes*	0.17	0.12	0.27

^a ^Total time includes screening plus the required 5 534 initial alignments between reference shapes and the query.

This increase in throughput is not surprising. For each conformer, we are only ever optimizing the overlay to the query for the 5 534 reference shapes. Also, the conformer database reference shape fingerprints are quite sparse, having only 1, 40, or 141 reference shapes set at minimum, average, or maximum, respectively. In contrast, ROCS requires optimizing the overlay of the query conformer to all fifteen million database conformers. As a means of comparison, CPU times required to search the dataset at Transform-Tanimoto values equal to 0.74 and 0.75 are also shown in Table 1. These timings indicate a two- and four-fold decrease, respectively, directly related to a substantial decline in the number of reference shapes considered during screening. This also suggests that each additional 0.01 decrease in the Transform-Tanimoto will increase the **AR **method CPU requirement by a factor of two.

**Figure 9:**
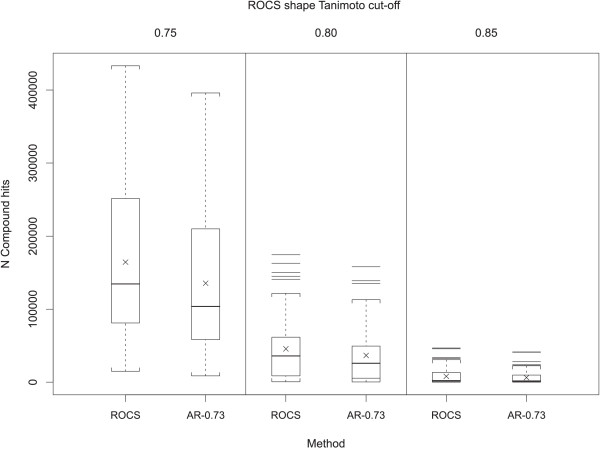
**ROCS versus AR-0.73 number of hits**. Box plots showing the distribution of the number of compounds found at 0.75, 0.80 and 0.85 shape Tanimoto cut-offs. Crosses represent the mean of each distribution. **AR-0.73 **retrieves fewer compounds than ROCS using the same cut-off.

**Figure 10:**
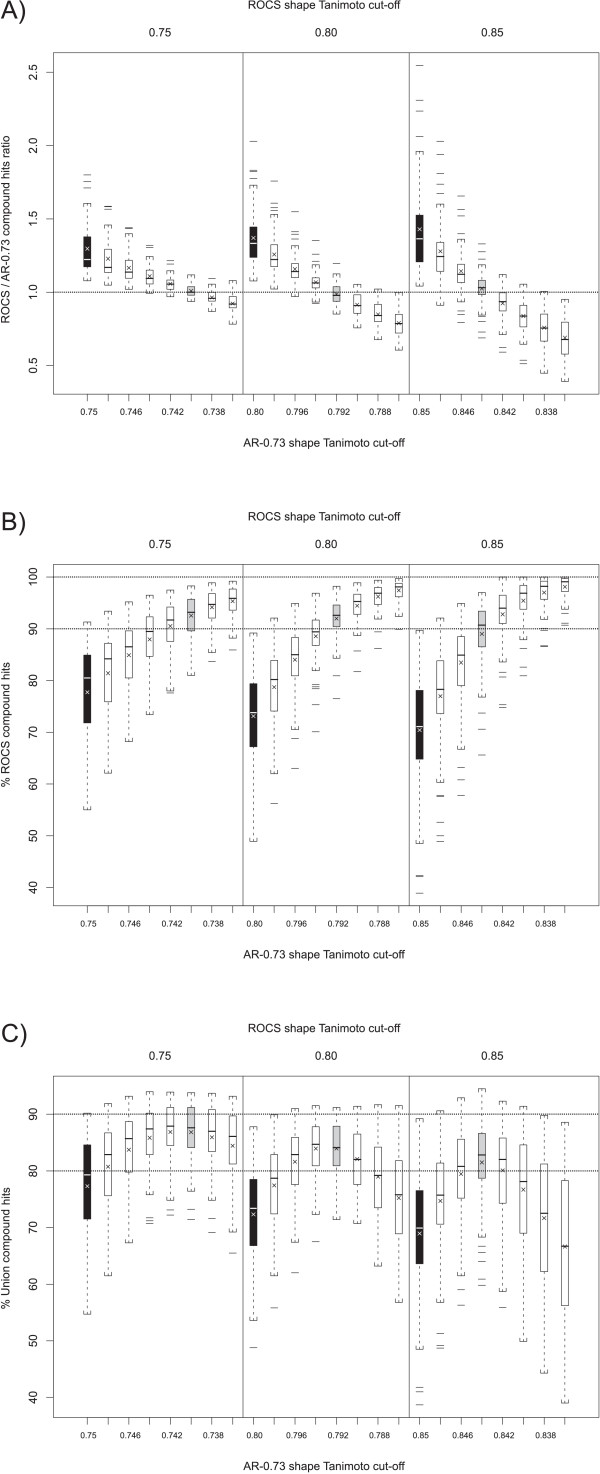
**ROCS versus AR at lower thresholds**. **AR-0.73 **is compared with ROCS at the shape Tanimoto cut-offs 0.75, 0.80, and 0.85, represented by black boxes, and also at lower **AR-0.73 **cut-offs until a similar number of hits are found, represented by grey boxes. **A) AR-0.73 **retrieves a similar number of hits (grey boxes cut the 1.0 ratio line) by decreasing the shape Tanimoto threshold by 0.01, 0.008 and 0.006 at 0.75, 0.80 and 0.85, respectively. **B) **Percentage of ROCS compounds found by **AR-0.73**. Around 90% of the ROCS compounds are found for hit lists of the same size (grey boxes). **C) **The union between **AR-0.73 **and ROCS hit lists is close to the maximum when hit lists have a similar size (grey boxes).

The overlap of **AR **using a 0.73 Transform-Tanimoto value (**AR-0.73**) and ROCS hit lists were examined to see if the **AR-0.73 **method produces results similar to ROCS using the shape Tanimoto similarity thresholds 0.75, 0.80, and 0.85. Figure 9 compares the count of compound hits using both methods. As shown in Table 2, **AR-0.73 **consistently produced ~20% fewer hits than ROCS on average, when using identical shape Tanimoto search thresholds. According to Figure 7, the **AR-0.73 **shape Tanimoto is, on average, 0.01 less than that resulting from an optimized alignment using ROCS. This suggests that a fairly small decrease in the **AR-0.73 **screening shape Tanimoto threshold, relative to that of ROCS, should bring the hit count, with similar alignment quality, into sync. Figure 10a shows how the relative count of hits grows as the **AR-0.73 **screening threshold is decreased, relative to ROCS. Figure 10a also shows that a similar count of query hits may be obtained at **AR-0.73 **shape Tanimoto values equal to 0.740, 0.792 and 0.844 for ROCS shape Tanimoto equal to 0.75, 0.80 and 0.85, respectively. Comparable hit counts, however, do not imply commonality of hit lists.

**Table 2:** Average compound hit list size resulting from querying the fifteen million conformer database with a single conformer

**Method**	**Shape Tanimoto Threshold**		
	
	**0.75**	**0.80**	**0.85**
*ROCS*	164 337.3	45 596.5	7 886.8
*AR-0.73*	135 505.9	36 670.3	6 267.1
*Ratio (AR-0.73/ROCS)*	82.5%	80.4%	79.5%

Figure 10b shows the ability of **AR-0.73 **to reproduce a growing percentage of the ROCS compound hit list as the **AR-0.73 **screening threshold is decreased, while keeping the ROCS screening threshold constant. As Figure 10c shows, however, that simply decreasing the **AR-0.73 **screening threshold only improves the union of the two compound hit lists to a point, after which diminishing returns sets in and the hit list overlap becomes worse. This result is expected considering decreasing the **AR-0.73 **screening threshold results in both ROCS hits missed by **AR-0.73 **and **AR-0.73 **hits that would be found by ROCS, if the ROCS screening threshold was not kept constant.

In each case, the **AR-0.73 **shape Tanimoto values 0.740, 0.792 and 0.844, originally highlighted as providing a similar number of compound hits, appear to also provide the best trade-off in maximizing the reproducibility of the ROCS hit lists for shape Tanimoto cut-off values equal to 0.75, 0.80 and 0.85. Using these shape Tanimoto values when comparing the two methods compound hit lists, one can expect the average similarity between the **AR-0.73 **and ROCS hit lists to be in the range of 81–87% (Table 3). The remaining 13–19% hit compounds that were not part of both hit lists belonged to one of the following four categories: hits found by **AR-0.73**, due to the use of a lower shape Tanimoto threshold, that would have been found by ROCS if the thresholds had been equal (5.7–6.1%); hits found by **AR-0.73 **but just missed by ROCS, due to finding a suboptimal solution during the maximization of the volume overlap or variation in the grid numeric precision (0.6–2.3%); hits missed by **AR-0.73**, but found by ROCS, due to the inability to find a reference shape in common (0.0–0.2%); and hits missed by **AR-0.73**, but found by ROCS, due to suboptimal volume overlap using alignment-recycling only, *i.e*., without overlap maximization optimization (6.7–10.1%). Regarding this last category, the **AR-0.73 **missed hits were only narrowly missed, with the missed hits having average shape Tanimoto values of 0.731, 0.787, and 0.840, just 0.009, 0.005, and 0.004 below the **AR-0.73 **similarity thresholds of 0.740, 0.792, and 0.844, respectively. This shows that even though the hit list intersection appears to decrease slightly with increasing shape Tanimoto value, the missed hits are increasingly proximate to the shape Tanimoto threshold.

**Table 3:** Comparison between ROCS and AR-0.73 hit lists when using reduced similarity thresholds for AR-0.73

**Compound hit list category**		**ROCS/AR-0.73 Threshold**		
		
		**0.75/0.740**	**0.80/0.792**	**0.85/0.844**
*Hits found by both methods*		86.8%	83.9%	81.5%
*Hits missed by ****AR-0.73***	*No common reference*	0.2%	0.0%	0.0%
	*With common reference*	6.7%	7.3%	10.1%
*Hits missed by ROCS*	*Threshold related*^a^	5.7%	7.5%	6.1%
	*Real miss*	0.6%	1.2%	2.3%

^a ^Missed hits artificially caused by using a lower **AR-0.73 **threshold than ROCS that would have been found, if the ROCS threshold had been the same as **AR-0.73**.

The observed correction for maximum overlap of **AR-0.73 **and ROCS hit lists as a function of shape Tanimoto appears to be linear. If this relationship holds across the entire range of ROCS shape Tanimoto values of 0.75 to 1.0, one could employ **Eq. 3 **to select the appropriate **AR-0.73 **shape Tanimoto cut-off to use for a desired ROCS shape Tanimoto value to achieve maximum overlap of results.

*ST*_*AR*-0.73 _= 1.04 * *ST*_*ROCS *_- 0.04

where *ST*_*AR*-0.73 _is the suggested optimum **AR-0.73 **shape Tanimoto value to use for a corresponding shape Tanimoto value, *ST*_*ROCS*_, in the range of 0.75 to 1.0.

## Discussion

The **AR-0.73 **method consistently reproduces ROCS results emphasizing that conformers with similar shapes tend to overlay to each other in a similar way. As such, overlay of two conformers, *A *and *B*, to a reference conformer, *R*, may generate an excellent approximation to the ideal alignment of conformers *A *and *B *by simply (re)using the alignments *AR *and *BR*. After finding a suitable set of reference shapes, the CPU cost to search for similar conformers across datasets of millions can be dramatically reduced. While efficient, the alignment-recycling method, **AR-0.73**, outlined in this work does have its limitations.

**AR-0.73**, in its current form, cannot be used for sub-shape comparison since global alignments are used. One can, however, readily imagine a subshape-based 3D fingerprint, much like dictionary-based 2D fingerprints. The implementation of such a method is beyond the scope of this work.

If a similar (enough) reference shape is not present when comparing two conformers, poor shape alignments may result, causing hits to be found by ROCS but missed by **AR-0.73**. If no reference shape is found to be in common, **AR-0.73 **cannot produce an alignment.

As the molecular size and flexibility increase, the number of required reference shapes is likely to increase dramatically to generate accurate shape overlays, which is probably the most important drawback of the **AR-0.73 **method. Reductions in the Design-Tanimoto can counter large increases in the number of reference shapes; however, in our experience, such a reduction in the Design-Tanimoto threshold, and concomitant reduction of the Transform-Tanimoto, can result in a reduction in the average quality of reproduction of the optimal overlay and an increased computational cost due to the consideration of additional conformers in alignment-recycling portion of the method. The overlay quality can be dramatically improved, in this situation, by slightly altering the methodology provided in this work to perform a post overlay optimization, using the near-optimal alignment-recycling overlay as a starting point for shape overlay optimization, providing substantial computational savings in the absence of such information. This proposed methodology extension may provide the means to apply aspects of the alignment-recycling method to larger and more flexible small molecules by eliminating the requirement that the recycled alignment reproduce the optimal alignment, thus allowing the Design-Tanimoto and Transform-Tanimoto thresholds to be (substantially) reduced.

Another drawback to **AR-0.73 **is that the primary computational expense is borne before any shape similarity searches are performed. For the fifteen million conformers used in this study, it took about four CPU years to compute the shape fingerprints using 64-bit 3-GHz Intel dual-core Xeon processors. Computational cost of the fingerprint generation is essentially recovered, however, after performing the same number of searches as there are reference shapes.

**AR-0.73**, while substantially reducing the CPU cost of shape similarity searching, adds concomitant demands on storing alignments to the reference shapes that must be available during the search. For the fifteen million conformer dataset, the (non-optimized) storage requirement for the fingerprints and rotational/translational information is 32 GB. If one is not careful, simultaneous access to this data can be a significant bottleneck.

If the **AR-0.73 **method is used with a dynamic database of conformers, additional computational costs can be envisioned. As new conformers are added, new reference shapes must be added dynamically whenever existing reference shapes cannot represent a new conformer. Addition of a new reference shape will require the precomputation step of comparing all existing database conformers to the new reference shape. After many new reference shapes are added (> 50% more of the initial total), a complete re-sampling of the reference shapes may be warranted to improve overall search performance through a reduction in the number of reference shapes. Also, for efficiency purposes, as conformers are deleted from the database, care must be taken to ignore reference shapes that no longer represent any database conformer to prevent unnecessary comparisons to a redundant reference shape.

With the above caveats in mind, the **AR-0.73 **method as described should be useful to speed the search of any 3D conformer dataset, regardless of size or flexibility. There should be no need to further modify the Transform-Tanimoto and Design-Tanimoto values of 0.73 and 0.75, respectively, to provide, *e.g*., complementary results to a ROCS search in the shape Tanimoto range of 0.75 – 1.0. The diverse reference shapes used in this work (see **Additional files**1 and 2) should be useful in helping create the initial reference shapes required to implement this method for arbitrary conformer databases. It is also reasonable to believe that the spirit of this methodology could be made to work using other shape searching packages besides ROCS.

Alterations to the **AR-0.73 **parameters, Transform-Tanimoto and Design-Tanimoto, may be made depending on the desired purpose. If one was only interested in use of this methodology as a shape search screen to dramatically reduce the number of conformers considered prior to shape overlay optimization and to provide reasonable starting points for overlay optimization, reduced values of the two parameters could be used, resulting in substantially fewer reference shapes and a significant reduction in the pre-computation cost. If one was only interested in reproduction of hit lists with shape Tanimoto values of 0.90 or greater, the Transform-Tanimoto could be increased closer to the Design-Tanimoto values, providing a further speed up in the shape search speed by reducing the number of conformers considered by alignment-recycling.

Overall, it appears clear that the **AR-0.73 **method, while an approximation to the optimal shape overlay, is very capable at routinely producing the vast majority of the ROCS results in a fraction of the CPU time.

## Conclusion

One of the main advantages of 3D overlay is that it allows visualization of the superimposed compounds and a better understanding of their similarity. Unfortunately, at the scale of large databases containing millions or billions of conformers, 3D alignment-based similarity searches are reserved to only entities with substantial computing capabilities and modeling resources. Even for such entities, it would be a major breakthrough to get nearly all of the desired alignments in just a couple of minutes using only a single CPU node. The alignment-recycling method described in this work shows promise in dramatically improving the speed of shape similarity searches of large databases through pre-computation of a small subset of shape overlays. Although the pre-computation requires significant computing resources, it is within the reach of modern, yet modest, computer clusters. The pre-computed transformation matrices to obtain the alignments with the subset can be effectively recombined to generate new alignments. Hit lists comparable to the Gaussian shape overlay optimizer ROCS can be obtained 100-times faster with only a small loss in alignment quality for smaller and relatively inflexible molecules. Suggested extensions and modifications to this methodology may prove handy in making 3D similarity a more tractable tool for use on large conformer databases.

## Methods

### Dataset

The subset of PubChem used for the analysis was extracted using the following protocol:

• Extract all the live records from the PubChem Compound [16] database

• Split mixtures into single covalent units

• Remove each structure not compliant with MMFF94s as implemented in OMEGA [20]

• Neutralize each ionic structure using a hydrogen atom, if chemically sensible

• Remove duplicate structures by comparing CACTVS stereo hash codes [23]

• Remove structures with incomplete stereochemistry (*i.e*., cis/trans double bonds or R/S stereo centers that are undefined)

• Remove structures with more than twenty-seven heavy atoms and more than five rotatable bonds

• Build the single conformer dataset using OMEGA 1.8.1 [20]

• Build the multiple conformer ensemble using OMEGA 2.0 Beta [20] and RMSD 1.0 Å spacing

### Gaussian shape overlay

The volume of a molecule is generally represented as the finite union of overlapping spheres, each one representing an atom. Although the most intuitive, the hard-sphere model involves complicated analytical expressions and gradient discontinuities. Grant and Pickup [24] overcame these problems by replacing the hard-sphere density function by a soft-sphere Gaussian equivalent, allowing rapid computation of molecular volumes. The smoothness of the Gaussian function and the simplicity of its derivatives greatly facilitate shape overlay optimizations [10]. Grant and Pickup algorithms are currently implemented in the OpenEye OEShape C++ toolkit [25]. The ROCS application is built using this toolkit. When we refer to ROCS, we are actually referring to the OEShape toolkit.

ROCS provides multiple conformer overlap determination methods. The *Grid *method is faster when many conformers are fit on a single reference conformer, but it treats all the heavy atoms as carbon, loosing overlap quality in some cases. For initial shape space coverage, we found the *Analytic *overlap method provided the best trade-off between the speed of the *Grid *overlap method and the precision of the *Exact *overlap method. For the alignment-recycling versus ROCS comparison we used the default ROCS *Grid *approach, as it is the fastest.

In this study, the atom radii used are Delphi radii, available from the OpenEye OEChem C++ library [26], and only non-hydrogen atoms are considered during shape comparisons. The shape similarity measure used is the Gaussian shape Tanimoto depicted in **Eq. 2**.

Alignment-recycling evaluates alignment-quality after each matrix multiplication through a single point shape Tanimoto computation. We used our own implementation of the ROCS *Grid *method. The results from our method are in essence identical to the results produced by ROCS (R^2 ^= 0.9998, SD = 0.00073, with N = 9 401 620 and maximum difference = 0.012).

### Diverse reference shape selection

The methodology for reference shape selection has been explained in great detail by Haigh *et al*. [15]. The dataset of conformers are clustered using a simple sphere exclusion algorithm. In the first step, a starting conformer is randomly selected as a reference shape. In the second step, all the conformers with a shape Tanimoto to the current reference shape greater than a pre-defined cut-off value (*i.e*., the "Design-Tanimoto" value) are assigned to the current reference shape cluster. For all the unassigned conformers, the shape Tanimoto to the most similar reference shape is stored. In the third step, the one conformer with the lowest stored similarity is selected as a new reference shape. The second and third steps are repeated until all conformers are assigned to a reference shape cluster. The Design-Tanimoto defines the resolution of coverage of the "shape space" of the dataset. The structure of the reference shapes is available in supporting information.

### Speed comparison

The test set from Oprea *et al*. [17] was extracted from the SD File available in the supporting information. Only 65 compounds met the PubChem subset selection criteria, *e.g*., for size and flexibility. We generated a 3D conformer model for each compound using OMEGA 2.0 Beta [20] and a RMSD cut-off of 1.0 Å. A single conformer was selected at random for each compound to perform the benchmark speed comparison. The conformer structure coordinates are available in supporting information. CPU time comparisons were performed using 64-bit 3-GHz Intel dual-core Xeon processors on the SuSE Enterprise 9.3 platform.

## Authors' contributions

FF wrote the first draft of the manuscript, developed the algorithms, and performed the testing experiments. EB participated in preparation of the PubChem dataset, design of experiments, and helped write the manuscript. YB participated in the conformer generation, gave advice on the design of experiments, and the writing of the manuscript. SHB supervised the work and provided critical review of the manuscript. All authors read and approved the final manuscript.

## Supplementary Material

Additional file 1 **diverse set of reference shapes**. MDL SD File containing 5 534 reference shapes with their 3D coordinates. The first 2 458 structures were selected using the single-conformer dataset. Click here for file

Additional file 2 **65 test compounds used to query the multi-conformer database**. MDL SD File containing 65 randomly selected 3D conformers, one for each compound. Click here for file
